# Myeloid-Derived Suppressor Cells and Therapeutic Strategies in Cancer

**DOI:** 10.1155/2015/159269

**Published:** 2015-05-19

**Authors:** Hiroshi Katoh, Masahiko Watanabe

**Affiliations:** Department of Surgery, Kitasato University School of Medicine, 1-15-1 Kitasato, Minami-ku, Sagamihara, Kanagawa 252-0374, Japan

## Abstract

Development of solid cancer depends on escape from host immunosurveillance. Various types of immune cells contribute to tumor-induced immune suppression, including tumor associated macrophages, regulatory T cells, type 2 NKT cells, and myeloid-derived suppressor cells (MDSCs). Growing body of evidences shows that MDSCs play pivotal roles among these immunosuppressive cells in multiple steps of cancer progression. MDSCs are immature myeloid cells that arise from myeloid progenitor cells and comprise a heterogeneous immune cell population. MDSCs are characterized by the ability to suppress both adaptive and innate immunities mainly through direct inhibition of the cytotoxic functions of T cells and NK cells. In clinical settings, the number of circulating MDSCs is associated with clinical stages and response to treatment in several cancers. Moreover, MDSCs are reported to contribute to chemoresistant phenotype. Collectively, targeting MDSCs could potentially provide a rationale for novel treatment strategies in cancer. This review summarizes recent understandings of MDSCs in cancer and discusses promissing clinical approaches in cancer patients.

## 1. Introduction

Chronic inflammatory stimuli are one of key risk factors for initiating and developing cancer. Indeed, the common pathological feature of chronic inflammation (e.g., chronic colitis) and solid cancer involves a massive infiltration of immune cells into the sites. The pathological changes in solid cancers include recruitment and modifying of various types of dysregulated immune cells and endothelial cells to form a tumor microenvironment [1]. A variety of chemokines and cytokines are produced by cancer cells and surrounding stromal cells and recruit leukocytes from the circulation to local sites according to their chemokine gradient. Cancer-associated fibroblasts (CAFs) constitute majority of the tumor stromal cells and play a critical role in tumor development [2]. Most of CAFs are also recruited from bone marrow via chemokine signaling as well as immune cells [3]. Cancer cells regulate and modify these immune cells to escape from host side immune system. A growing body of evidence supports that cancer initiation and progression essentially depend on escape from host immunosurveillance. Immune evasion involves a shift of immune responses, including imbalance in Th1/Th2 responses and enhancement of immunosuppressive cells such as myeloid-derived suppressor cells (MDSCs), regulatory T cells, M2 macrophages (tumor-associated macrophages), and type 2 NKT cells. MDSCs are a heterogeneous population of immune cells characterized by the ability to suppress cytotoxic functions of T cells and NK cells [4]. MDSCs originate from myeloid progenitor cells and are thought to be immature cells that do not differentiate into granulocytes, macrophages, or dendritic cells (DCs). Pathological conditions such as infection, trauma, autoimmune diseases, and cancer trigger expansion of MDSCs in bone marrow and spleen. MDSCs then accumulate in the peripheral blood, tumor, lymphoid organs, and parenchymal organs. In the past decade, MDSCs have been thought essential especially in solid cancers and one of key drivers of not only cancer-associated immune evasion but also tumor progression and metastasis by establishing tumor microenvironment [5]. Indeed, the number of circulating MDSCs in the peripheral blood correlates well with clinical cancer stage and metastatic tumor burden in patients [6, 7]. MDSCs also play a key role in gaining chemoresistant phenotype in cancer [8, 9]. Therefore, targeting MDSCs would be promising treatment option for patient with cancer. This review summarizes and discusses the recruitment mechanisms and immunosuppressive functions of MDSCs and the potential strategies to target cancer-associated MDSCs.

## 2. Phenotypes of MDSCs in Cancer

**Figure 1:**
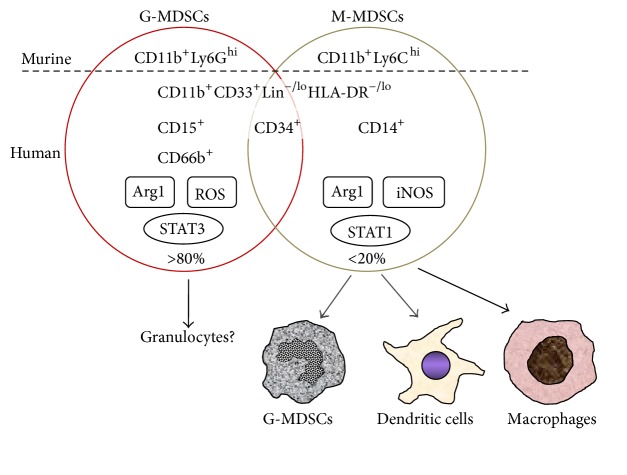
Surface markers and suppressive mechanisms of MDSCs. In murine cancer models, G-MDSCs (CD11b^+^Ly6G^hi^Ly6C^lo^) and M-MDSCs (CD11b^+^Ly6C^hi^Ly6G^−^) can be discriminated by the cell surface markers. However, identifying human MDSCs is still challenging because of their phenotypic heterogeneity and the absence of cognate surface markers in mice. Generally, human MDSCs can be defined as CD11b^+^CD33^+^Lin^−/lo^HLA-DR^−/lo^ and further divided to CD15^+^ or CD66b^+^ G-MDSCs and CD14^+^ M-MDSCs. A substantial population shows both CD15 (or CD66b) and CD14 negative, suggesting that G-MDSCs and M-MDSCs are not completely distinct population. M-MDSCs can differentiate mature dendritic cells and macrophages or putatively G-MDSCs.

MDSCs are composed of heterogeneous immature myeloid cells that arise from bone marrow progenitor cells, at different stages of differentiation from early myeloid cells to more differentiated macrophages, granulocytes, or dendritic cells. MDSCs accumulate in tumor tissues and in the peripheral lymphoid organs. MDSCs are also found to infiltrate the spleen and liver [4]. Circulating CD11b^+^Gr1^+^ cells are arrested and accumulate in the splenic marginal zones and migrate to the red pulp and proliferate [10, 11], suggesting that CD11b^+^Gr1^+^ cells in peripheral blood may represent both proliferated MDSCs and precursors for MDSCs. In mice, MDSCs are broadly characterized by CD11b^+^Gr-1^+^, and MDSCs are classified to two subsets as either granulocytic (polymorphonuclear) MDSCs (G-MDSCs) or mononuclear MDSCs (M-MDSCs) (Figure 1). G-MDSCs are defined as CD11b^+^Ly6G^hi^Ly6C^lo^ and M-MDSCs as CD11b^+^Ly6C^hi^Ly6G^−^ [4]. Although the pattern of G-MDSC and M-MDSC subsets differs between tumors and organs, over 80% of MDSCs are G-MDSCs, whereas less than 10% of MDSCs are M-MDSCs in most of experimental models [12–14]. However, substantial neutrophils also express both CD11b and Ly6G, causing difficulty to discriminate G-MDSCs from neutrophils. In addition to reactive oxygen species (ROS) and arginase 1 (Arg1), M-CSFR and CD244 have been proposed as phenotypes of G-MDSCs [15].

In human, phenotypes of human MDSCs are yet to be clarified and much more complicated with phenotypic diversity and heterogeneity. Circulating CD33^+^ cells, CD33^+^HLA-DR^−^, or CD33^+^HLA-DR^−^Lin^−^ are described to be MDSCs in patients with renal cell cancer, colorectal cancer, or hepatocellular carcinoma, respectively [16–18]. CD11b^+^CD33^+^ cells are reported as MDSCs in peripheral blood of patients with non-small cell lung cancer [19]. Yu et al. suggested CD45^+^CD13^+^CD33^+^CD14^−^CD15^−^ MDSCs in tumors and peripheral blood of breast cancer patients [20]. In general, human MDSCs are defined as CD11b-positive, CD33-positive, HLA-DR-negative or low, and lineage markers (Lin) (CD3, CD14, CD19, CD56)-negative [11]. Zhang et al. described circulating CD11b^+^CD33^+^HLA-DR^−^Lin^−/lo^ MDSCs and their detailed profiles as CD13^hi^CD39^hi^CD115^lo^CD117^lo^CD124^lo^PD-L1^lo^CD14^−^CD15^−^CD66b^−^ in patients with colorectal cancer [21]. Similarly to mice, human MDSCs can be phenotypically classified as granulocytic and monocytic population (Figure 1). G-MDSCs express a granulocytic marker CD15 or CD66b in addition to CD11b and CD33 in head and neck cancer, non-small cell lung cancer, pancreas cancer, bladder cancer, renal cell cancer, and breast cancer [22–27]. On the other hand, human M-MDSCs have been described as CD14^+^HLA-DR^−^(CD33^+^Lin^−^) in hepatocellular carcinoma, metastatic prostate cancer, renal cell cancer, and malignant melanoma [28–31]. M-MDSCs have been generally described as CD11b^+^CD14^+^HLA-DR^lo^ or CD11b^+^CD14^−^CD15^−^ (or CD66b^−^) [22–25]. Vasquez-Dunddel et al. defined M-MDSCs in tumors, draining lymph nodes, and peripheral blood as CD11b^+^CD33^+^CD14^+^HLA-DR^−/lo^CD34^+^Arg1^+^ROS^+^ in head and neck squamous cancer [32]. Numerous combinations of these markers have been reported in human solid cancers [33].

## 3. Mechanism of MDSC-Mediated Immunosuppression

Immunosuppression of cytotoxic T cells and NK cells is a hallmark of MDSCs. Direct cell-to-cell contact is required for such immunosuppressive activities of MDSCs [4]. Numerous preclinical studies have showed the mechanisms of MDSC-mediated immune suppression. MDSCs suppressive activity against T cells is associated with L-arginine metabolism. L-arginine is a substrate for inducible nitric oxide synthase (iNOS) and arginase 1 (Arg1). Arg1 and reactive oxygen species (ROS) are upregulated in activated G-MDSCs, while Arg1 and iNOS are highly expressed in activated M-MDSCs. The upregulation of either Arg1 or iNOS results in L-arginine shortage, leading to consequent inhibition of T cell proliferation through multiple mechanisms such as reduction of CD3 *ζ*-chain expression and IFN-*γ*/IL-2 secretion by T cells [4, 13, 34, 35]. High levels of ROS in G-MDSCs can induce nitrosylation of the T cell receptor (TCR) during direct cell-to-cell communication, which contributes to the inhibition of antigen-specific T cell activation [13, 36, 37]. ROS production by G-MDSCs is known to be induced by several tumor-derived factors such as TGF*β*, IL-6, IL-10, and GM-CSF [4]. The suppressive function of G-MDSCs depends on Arg1 and ROS, whereas that of M-MDSCs requires signal transducer and activator of transcription 1 (STAT1) and iNOS [38]. In activated G-MDSCs, STAT3 is highly activated, which results in increased expression levels of ROS via upregulation of NADPH oxidase (NOX2) but not NO production. On the other hand, STAT1 and iNOS are highly upregulated in M-MDSCs, resulting in increased levels of NO but not ROS production [4]. In addition, STAT6 signaling pathway is involved in upregulation of Arg1 and TGF-*β* through activation of IL-4 and IL-13, leading to immunosuppressive activity [39, 40]. However, the immunosuppressive mechanisms overlap between G-MDSCs and M-MDSCs in human cancers. iNOS is also upregulated in G-MDSCs in a variety of human cancer [24, 41, 42]. CD14^+^HLA-DR^−/lo^ M-MDSCs express NADPH oxidase component gp91 (phox) and produce high level of ROS in human non-small cell lung cancer [43]. These M-MDSCs inhibit T cell proliferation and IFN-*γ* secretion in a cell-contact-dependent manner.

Another mechanism of immunosuppressive activity of MDSCs against T cells is cysteine deprivation. Cysteine is essential for T cell activation but cannot be synthesized by T cells. Antigen-presenting dendritic cells and macrophages can deliver cysteine by converting methionine and cystine to cysteine [44]. MDSCs also import extracellular cystine for converting it to cysteine but they do not export cysteine, leading to lack of cystine for dendritic cells and macrophage [45]. Recently, another subset of MDSCs, cancer-induced fibrocytes, is proposed. This novel subset of MDSCs shows phenotypic and functional characters of fibrocytes but mediates immune suppression by inhibiting T cell proliferation via indoleamine oxidase (IDO) in human metastatic pediatric sarcomas [46].

MDSCs can suppress the cytotoxic activity of NK cells and their IFN-*γ* production [47]. CD14^+^HLA-DR^−/lo^ M-MDSCs inhibit NK cell cytotoxicity and cytokine secretion in a cell-contact-dependent manner in human hepatocellular carcinoma [48]. The inhibition of NK cells is independent of arginase activity. On the other hand, MDSC-mediated immunosuppressive activities against NK cells are mainly dependent on the NKp30 on NK cells. Furthermore, MDSCs can skew macrophage-derived cytokine profiles from type 1 to type 2 putatively through Toll-like receptor 4 signaling pathway [49]. Indeed, increased MDSC levels in peripheral blood and tumor are closely associated with the infiltration of CD163^+^ M2 macrophages in human esophageal cancer [50].

## 4. Expansion and Trafficking of MDSCs

Although mechanisms of induction and trafficking MDSCs are still elusive, multiple tumor-derived factors are suggested in preclinical models and cancer patients. These tumor-derived factors include granulocyte-macrophage colony-stimulating factor (GM-CSF), granulocyte colony-stimulating factor (G-SCF), macrophage colony-stimulating factor (M-CSF), IL-1*β*, IL-6, VEGF, IL-13, IL-10, prostaglandin E_2_ (PGE_2_), TNF, bombina variegate peptide 8 (Bv8, also known as prokineticin 2 or Prok2), and stem cell factor (SCF) [51–61]. Most of these tumor-derived factors activate signaling pathways which involve STAT3 or Janus kinase (JAK) in myeloid precursor cells [62]. Among these factors, GM-CSF and IL-6 may be most powerful in inducing MDSCs from bone marrow hematopoietic progenitors [63]. In addition to bone marrow, spleen is another source of MDSCs. Indeed, GM-CSF induces the KIT^+^ MDSC precursors in spleen of tumor-bearing mice [64]. GM-CSF is also required for recruitment of MDSCs to the tumor microenvironment [64, 65]. The STAT3 activation in myeloid precursor cells enhances MDSC proliferation and expansion in bone marrow and also promotes the production of calcium binding proteins S100A8 and S100A9 which form heterodimers and inhibit differentiation to dendritic cells, contributing expansion and accumulation of MDSCs [66]. S100A8/A9 bind to cell surface glycoprotein receptors on MDSCs and promote MDSC activation and migration through the NF-*κ*B signaling pathway. MDSCs also synthesize and secrete S100A8/A9, resulting in an autocrine feedback loop that sustains accumulation of MDSCs [67]. Bv8 mobilizes immature myeloid cells from hematopoietic progenitor cells, and anti-Bv8 treatment decreases mobilization of CD11b^+^Gr1^+^ cells from bone marrow [61]. Tumor-derived PGE_2_ promotes differentiation of CD11b^+^Gr1^+^ cells from bone marrow stem cells through EP2 signaling [59]. PGE_2_ stimulates MDSCs to produce high levels of Arg1 and iNOS in tumor and spleen through EP4 signaling pathway, leading to T cell suppressive functions [35, 68]. In human ovarian cancer, positive feedback loop between PGE_2_ and cyclooxygenase-2 (COX-2) facilitates the differentiation of human CD1a^+^ DCs toward stable M-MDSCs (CD14^+^CD11b^+^CD33^+^CD34^+^) [57]. CCAAT-enhancer-binding protein *β* (C/EBP*β*) also contributes to MDSCs expansion and activation of the immunoregulatory activity of MDSCs [69]. Additionally, human hepatic stellate cells (HSCs) may have capacity to transform monocytes in peripheral blood into MDSCs in a CD44-dependent manner [70].

As aforementioned, GM-CSF promotes migration of MDSCs to tumor. Several chemokine systems have been suggested to be involved in recruitment of MDSCs to the primary cancer or the premetastatic niche. Tumor-derived CXCR2-ligands (IL-8, CXCL1, CXCL2, and CXCL5) attract MDSCs to the tumor microenvironment [71–73] and inhibition of CXCR2 profoundly suppresses Gr-1^+^ leukocyte migration into tumor [74]. CXCR2 is expressed on most of circulating G-MDSCs but not on M-MDSCs and is prerequisite for G-MDSCs to be recruited to tumor microenvironment [12].

Tumor-derived inflammatory mediator PGE_2_ promotes CXCL12 production via EP2/4 receptor signaling and thus accumulates CXCR4-expressing MDSCs in human ovarian cancer [75]. On the other hand, CCR2^+^ M-MDSCs are recruited by tumor-derived CCL2 in mice and human [76–78]. S100A8 and S100A9 not only activate MDSCs but also attract MDSCs by binding receptor for advanced glycation end products (RAGE) on MDSCs. Recruited MDSCs secrete S100A8/A9, resulting in further accumulation of MDSCs in human breast cancer [8].

## 5. Other Functions of MDSCs in Cancer

G-MDSCs promote tumor angiogenesis via secreting Bv8 which is regulated by STAT3 activation [79]. STAT3 activation also induces the secretion of VEGF and bFGF from MDSCs [80]. MDSCs also promote tumor-associated angiogenesis by secreting matrix metalloproteinase-9 (MMP-9) [54]. Accumulating evidences show that cancer cells disseminate at early phase of tumor progression. MDSCs facilitate cancer cell invasion and intravasation by secreting multiple proteolytic enzymes including MMPs which are necessary for extracellular matrix degradation and disruption of endothelial cadherins, adhesion proteins or basement membrane of vessels [81–83]. Epithelial-mesenchymal transition (EMT) is one of the steps for dissemination of cancer cells. When cancer cells undergo EMT, cancer cells lose epithelial markers and gain mesenchymal phenotypes. G-MDSCs induce EMT in cancer cells using TGF-*β*, epidermal growth factor (EGF), and hepatocyte growth factor (HGF) [73]. On the other hand, MDSCs also contribute to mesenchymal-epithelial transition (MET) of cancer cells by secreting versican [84]. This MDSC function supports cancer cells to colonize at metastatic niche.

MDSCs interact with cancer cells and induce microRNA101 (miR-101) expression. miR-101 subsequently decreases the corepressor gene C-terminal binding protein-2 (CtBP2), which results in increased cancer cell stemness in ovarian cancer [85].

On the contrary to prometastatic functions of MDSCs, Gr-1^+^ myeloid cells generate a metastasis-resistant microenvironment in distant organs through the induction of thrombospondin-1 (Tsp-1) by tumor-derived prosaposin in mice bearing metastasis-incompetent tumors [86]. This may represent not only heterogeneity of MDSCs but also functional plasticity of MDSCs, and the activities of MDSCs might depend on a type of primary tumor. In addition, CD11b^+^Ly6G^+^ myeloid-derived cells differentiate into tumor-entrained neutrophils (TENs), which inhibit formation of premetastatic niche in the lungs [87]. It is unclear whether TENs represent differentiated stage of MDSCs or independently matured cells.

## 6. Clinical Therapeutic Strategies Targeting MDSCs in Cancer

**Figure 2:**
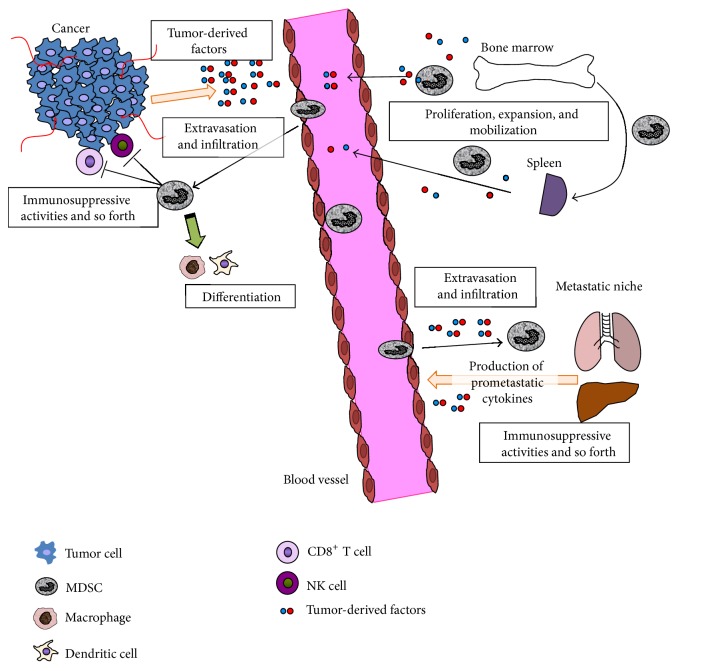
Schema of MDSC expansion and recruitment machinery. Tumor-derived factors (e.g., GM-CSF, IL-6, S100A8/A9, and PGE_2_) promote proliferation, expansion, and mobilization of MDSCs from bone marrow hematopoietic progenitor cells, and tumor-derived chemokines (e.g., CXCL1/2 and CXCL12) recruit MDSCs to primary tumor and metastatic niche according to their chemokine gradients. Bone marrow-derived MDSC precursors are also arrested at marginal zone of spleen and migrate to the red pulp and proliferate. Recruited MDSCs support tumor progression by the immunosuppressive activities against cytotoxic CD8^+^ T cells and NK cells. M-MDSCs can differentiate to mature nonsuppressive dendritic cells or type 1 macrophages. MDSCs also facilitate tumor-associated angiogenesis and epithelial-mesenchymal transition of cancer cells, which results in invasion and extravasation. On the other hand, MDSCs can contribute to mesenchymal-epithelial transition at the metastatic niche.

Most of preclinical and clinical evidences indicate the association of MDSCs with poor prognostic outcome as well as immunosuppressive effect in tumor microenvironment both in primary and metastatic tumor regardless of the heterogeneity of MDSCs particularly in human cancer patients. The cancer-supportive activities provide a rationale for therapeutic approaches to target cancer-associated MDSCs. The strategies targeting MDSCs would be roughly divided into two approaches: (1) directly harnessing the mechanisms of MDSC expansion, recruitment, or immunosuppressive processes (Figure 2) to target the protumorigenic/prometastatic activities of MDSCs and (2) sensitizing other anticancer agents through suppressing MDSC accumulation.

Several potential strategies for targeting MDSCs can be proposed for each step of MDSC accumulation and the activities (Figure 2). First potential treatment strategy is inhibiting tumor-derived or MDSC-derived factors which expand and mobilize MDSCs from bone marrow or spleen. Several neutralizing antibodies or inhibitors against tumor-derived factors or those receptors such as GM-CSF, GM-CSF receptor (GM-CSFR) [64], M-CSF, M-CSF receptor (M-CSFR) [88, 89], G-CSF [90], VEGF-A [91], or stem cell factor (SCF or KIT) [60] have been reported to inhibit MDSC expansion or mobilization. However, anti-VEGF-A monoclonal antibody, bevacizumab, could not reduce the accumulation of MDSCs in human renal cell cancer [92]. Anti-IL-6 receptor monoclonal antibody neutralizes tumor-derived IL-6 and thus suppresses expansion of cancer-associated MDSCs [93]. Also, directly inhibiting MDSC proliferation from hematopoietic precursor cells in bone marrow or spleen would be an approach to suppress MDSC expansion as well as decreasing circulating MDSCs. Some chemotherapy drugs such as gemcitabine and 5-fluorouracil decrease circulating MDSCs [94, 95]. However, one study shows that combination of gemcitabine and capecitabine does not affect the levels of MDSCs in patients with advanced pancreatic cancer [96]. An aminobisphosphonate zoledronic acid (zoledronate) reduces mobilization of MDSCs through suppressing bone marrow progenitor-derived and tumor-derived MMP-9 [97, 98] although one study reports that zoledronic acid impairs tumor-associated macrophage proliferation but not MDSCs [99]. Sunitinib is a multikinase inhibitor which inhibits VEGFR1-3, platelet-derived growth factor receptor- (PDGFR-) *α* and *β*, stem cell factor receptor (c-Kit), FLT3, and RET [100]. Sunitinib also reduces MDSC proliferation [23, 101]. Vemurafenib is a highly specific inhibitor of mutant B-RAF^V600E^ and suppresses the release of tumor-derived soluble factors involved in MDSC generation [102]. Peroxisome proliferator-activated receptor-*γ* (PPAR*γ*) is an anti-inflammatory molecule expressed in myeloid-lineage. Dominant-negative PPAR*γ* expression in myeloid cells reduces expansion of CD11b^+^Ly6G^+^ population [103].

Of course, it is promising to inhibit accumulation of MDSCs by targeting aforementioned tumor-derived factors or tumor-related inflammatory mediators or by using chemotherapeutic agents or monoclonal antibodies which are thought to suppress expansion of MDSCs. However, some limitations should be considered particularly in clinical studies, that is, “cause or result” issue. The tumor-derived factors or inflammatory mediators can facilitate tumor progression by themselves. And the chemotherapeutic agents or monoclonal antibodies which are thought to inhibit MDSC accumulation also target cancer cells themselves, consequently leading to reduction of MDSCs. Accordingly, the attenuation of MDSC accumulation may, at least partly, reflect the reduction of the tumor burden caused by the therapeutic agents.

A second therapeutic strategy would be inhibiting MDSC recruitment from the circulation to the tumor microenvironment or to the peripheral lymphoid organs. Tumor-derived CXCR2-ligands, CXCL1, CXCL2, and CXCL5, have been known to promote recruitment of MDSCs to the site of tumor or to the premetastatic niche [12, 71–73, 104]. Since CXCR2 is mainly expressed on G-MDSCs, CXCR2 inhibition may impair the recruitment of G-MDSCs [12]. Accordingly, it is warranted to clarify the organ-specific preferences of MDSC types. Other antagonists or inhibitors for chemokine systems such as anti-CXCR4 antibody and CCL2 neutralizing antibody would have potential to inhibit MDSC accumulation [4, 75, 105]. CSF1R antagonist can suppress the infiltration of MDSCs and tumor-associated macrophages (TAMs) to the site of tumor [88, 89]. Recently, one study suggested MDSC-specific peptides (H6 and G3) and generated peptide-Fc fusion proteins (peptibodies) which bind and affect both G-MDSCs and M-MDSCs [106]. These peptibodies specifically depleted MDSCs without affecting proinflammatory immune cell types such as mature dendritic cells. Since these peptibodies target only MDSCs, they have potential to solve aforementioned “cause or result” issue of MDSC-targeting therapies.

Thirdly, therapeutic strategy would involve directly blocking suppressive activities of MDSCs. Cyclooxygenase-2 (COX-2) inhibitors suppress activation of MDSCs through CCL2, CXCL12, or PGE_2_ inhibition and increase cytotoxic T lymphocytes (CTLs) [11, 75, 107]. Phosphodiesterase-5 (PDE-5) inhibitors (sildenafil, tadalafil, and vardenafil) are currently applied for clinical use in nonmalignant diseases (e.g., pulmonary hypertension). PDE-5 inhibitors increase infiltration of activated CTLs into tumor and tumor-induced T cell proliferation [108]. PDE-5 inhibitors restore *ζ*-chain expression in TCR by decreasing MDSCs [109, 110]. Importantly, PDE-5 inhibitors downregulate expression of Arg1, iNOS, and IL-4*α* in MDSCs, which results in restoration of cytotoxic activities of T cells [108]. Synthetic triterpenoid C-28 methyl ester of 2-cyano-3,12-dioxooleana-1,9,-dien-28-oic acid (CDDO-Me, bardoxolone methyl) abrogates the immunosuppressive activities of MDSCs and restored immune responses in both preclinical murine model and patients with renal cell carcinoma [111].

A fourth strategy would be promoting the differentiation of MDSCs into mature, nonsuppressive cells. All-trans retinoic acid (ATRA), a vitamin A derivative, induces MDSC differentiation into mature myeloid cells by upregulating glutathione synthase in MDSCs [112]. Removal of immature myeloid cells from DC fractions by ATRA restored the ability of DCs to stimulate antigen-specific T cell activity [113]. In patients with metastatic renal cell carcinoma, ATRA administration decreased circulating CD33^+^HLA-DR^−^Lin^−/low^ MDSCs, which leads to improved myeloid/lymphoid DC ratio and antigen-specific T cell response [114]. Immune suppressive CD34^+^ progenitors (MDSCs) are increased in peripheral blood and tumors of patients with head and neck squamous cell cancer (HNSCC). These CD34^+^ MDSCs isolated from peripheral blood of HNSCC patients can be differentiated into phenotypically and functionally DC-like cells* in vitro*, and 1,25-dihydroxyvitamin D3 (1,25(OH)_2_D3) accelerates differentiation of MDSCs into the DC-like cells [115]. Indeed, 25-hydroxyvitamin D3 reduced the circulating CD34^+^ MDSCs in HNSCC patients which resulted in increased levels of plasma IL-12 and IFN-*γ* and T cell proliferation [116]. Although 25-hydroxyvitamin D3 alone failed to improve clinical outcome [116], it may have a potential to sensitize other chemotherapeutic agents by reducing MDSCs. One study shows that tumor necrosis factor-*α* (TNF-*α*) blocks differentiation and augments suppressive activity of MDSCs in chronic inflammatory settings and that administration of a TNF-*α* antagonist, etanercept, reduces MDSCs' suppressive activity and promotes their maturation into dendritic cells and macrophages [117]. However, this study is based on chronic inflammation model and remains to be confirmed in tumor-bearing model as well.

Several preclinical studies have shown that MDSCs play a critical role in refractoriness against anticancer drugs such as bevacizumab, cyclophosphamide, anthracycline, and sunitinib [6, 8, 9, 118, 119] (Table 1). Therefore, targeting MDSCs would lead to sensitizing these drugs. An anti-VEGF monoclonal antibody, bevacizumab, and a multikinase inhibitor, sunitinib, upregulate Bv8 production from MDSCs, unexpectedly leading to tumor-associated angiogenesis. Indeed, anti-Bv8 antibody treatment restores antiangiogenic effects and shows additive cytotoxic effect on those of anti-VEGF antibody therapy [118]. Anthracycline and cyclophosphamide (AC) regimen enhances production of tumor necrosis factor-*α* (TNF-*α*) from tumor stromal cells and endothelial cells. Consequently, TNF-*α* boosts CXCL1/2 production by cancer cells through NF-*κ*B signaling. CXCL1/2 promote recruitment of G-MDSCs which secrete S100A8/9, which results in amplifying the CXCL1/2-S100A8/9 loop and causing increased cancer cell survival, metastasis, and chemoresistance [8]. Accordingly, CXCR2 blockade may impair this vicious cycle. Interestingly, tumor-derived G-CSF induced MDSCs may contribute to radioresistant feature in uterine cervical cancer [120]. Anti-Gr-1 neutralizing antibody or MDSC depletion by splenectomy restored radiosensitivity in cervical cancer xenografts. Collectively, several chemotherapeutic agents or radiotherapy unexpectedly stimulates secretion of tumor-derived factor, which leads to expansion of MDSCs critically causing refractoriness against the treatments. To break this vicious cycle by inhibiting recruitment of MDSCs may sensitize these chemotherapeutic agents or radiotherapy and prevent the refractoriness against the anti-cancer therapies.

A variety of promising combination therapies targeting MDSCs have been proposed. Combination of antigen-specific peptide and ATRA significantly increases and prolongs antigen-specific T cell response [121]. In patients with extensive stage small cell lung cancer, ATRA improved the immune response to vaccination by significant reduction of CD33^+^HLA-DR^−^Lin^−/low^ MDSCs and consequent restored antigen-specific T cell response [122]. Of note, ATRA administration does not cause significant toxicity [114]. HER2/neu is a self-antigen with poor immunogenicity which is overexpressed in human cancer cells such as breast cancer. Gemcitabine treatment with HER2/neu vaccine and antiglucocorticoid-induced tumor necrosis factor receptor (TNFR) family-related receptor (GITR) antibody is effective in HER2/neu-positive tumor [123], in which gemcitabine attenuates the tumor immune suppression through dramatic reduction of MDSCs. Accordingly, combination therapy of immunotherapies and gemcitabine would be a promising treatment option. Sunitinib, a small molecule inhibitor of multiple receptor tyrosine kinases, reduces MDSCs in peripheral blood of patients with renal cell carcinoma [101]. The addition of sunitinib to low-dose radiation additionally suppressed tumor growth in glioblastoma [124]; however, the mechanisms of the tumor suppressive effect in this combination therapy are still unclear. The programmed death 1 (PD1) receptor is an important immune checkpoint which is expressed on T cells, and PD1 signaling inhibits T cell receptor-mediated activation [125]. Recently, clinical evidences have shown limited efficacy of anti-PD1 monoclonal antibody in a minority of cancer patients. Rhabdomyosarcoma attenuates the efficacy of anti-PD1 therapy by inducing expansion of CXCR2^+^CD11b^+^Ly6G^high^ G-MDSCs in preclinical model [126]. Anti-CXCR2 monoclonal antibody therapy improved significant antitumor effects of anti-PD1 treatment. These findings suggest that inhibition of CXCR2^+^ G-MDSC tumor trafficking may sensitize and enhance anti-PD1 monoclonal antibody therapy in other cancers. Collectively, targeting MDSCs is a promising strategy to improve or increase immunotherapies against cancers.

One interesting preclinical study indicates the application of MDSCs as a vehicle to deliver radioisotope-coupled attenuated variant of* Listeria* monocytogenes to tumor sites [127]. This may be applicable to other anticancer agents.

On the other hand, as various studies show that circulating MDSC levels are correlated with tumor stages [21, 128], MDSCs would be a good marker for a potential relapse of tumor or a surrogate marker which represents treatment efficacy. For instance, an elevated activity of circulating MDSCs indicated by increased NO production is suggested to be an early marker of incomplete treatment and a potential relapse of cancer in non-small cell lung cancer [129]. Also, MDSC levels in peripheral blood may predict chemotherapy failure in patients with pancreatic adenocarcinoma [27]. The sensitivity and specificity circulating MDSC levels or the activation markers are still elusive.

## 7. Summary

Immune evasion is a hallmark of cancer, and cancer-induced MDSCs play an essential role in tumor progression through tumor-associated immunosuppression. Therefore, it is a promising approach to break the vicious cycle in which MDSCs are expanded and recruited to support tumor progression. Several approaches have been sought, for example, inhibition of tumor-derived factors, suppression of generation and expansion of MDSCs from hematopoietic progenitors, blockade of MDSC trafficking and infiltration, harnessing immune suppressive activities of MDSCs, or facilitating differentiation of MDSCs into mature nonsuppressive cells. Furthermore, targeting MDSCs may enhance and sensitize the effects of chemotherapies, vaccine therapies, or radiotherapies. However, MDSCs are composed of heterogenous populations which are probably in different stages of differentiation and may have different activity in individuals with cancer. Importantly, markers of MDSCs in human are much more complicated than in mice, leading to further heterogeneity and difficulty. MDSC biology including these limitations remains to be elucidated for development of MDSC-targeting therapy, patient selection, and decision of treatment strategy.

## Figures and Tables

**Table 1:** MDSCs-mediated refractoriness to chemotherapy or radiotherapy.

Treatment	Mechanisms	References
Anti-VEGF antibody (bevacizumab)	Bv8 production in MDSCs	[118]

Anthracycline/cyclophosphamide	S100A8/9 production in accumulated MDSCs via secretion of CXCL1/2 by cancer cells	[8]

Tyrosine kinase inhibitor (sunitinib)	Bv8 production in MDSCs pSTAT5-dependent pathway via GM-CSF production	[118, 119]

Radiotherapy	G-CSF-mediated MDSC accumulation	[120]
